# Successful treatment of complex malignant bowel obstruction with EUS-guided enterocolostomy (with video)

**DOI:** 10.1097/eus.0000000000000186

**Published:** 2026-07-15

**Authors:** Sa Fang, Xuegang Guo, Mengmeng Zhang, Jie Hao, Yupeng Shi

**Affiliations:** 1 Department of Gastroenterology, Honghui Hospital, Xi’an Jiaotong University, Xi’an, Shaanxi Province, China; 2 Department of Hepatobiliary Surgery, the First Affiliated Medical College of Xi’an Jiaotong University, Xi’an, Shaanxi Province, China.

Primary or metastatic malignant tumors and their antitumor treatment can cause a bowel obstruction of the Treitz ligament called malignant bowel obstruction (MBO), which is among the common late clinical symptoms of peritoneal metastasis of colorectal cancer, with a reported incidence of 10%–28%.^[1]^ Its pathophysiological mechanism involves multiple factors, including local tumor invasion and compression, intestinal adhesions caused by scattered abdominal nodules, inflammatory reactions within the abdominal cavity, and neuromodulation disorders.^[2]^

MBO associated with peritoneal metastasis from rectal cancer presents with multisegmental obstructions, complex and diverse symptoms, extensive abdominal metastases, and even cachexia. These features are often difficult to treat surgically, making MBO a major therapeutic challenge in colorectal surgery.^[2]^ For MBO patients who cannot be surgically treated, the average survival time of these patients is 4–5 weeks.^[3]^ In contrast, for patients who undergo intestinal bypass surgery, the 6-month survival rate can reach 62%. Furthermore, the treatment of the primary disease after surgery can prolong the median survival time.^[4]^

The use of a lumen-apposing metal stent (LAMS) has revolutionized the treatment of gastrointestinal diseases that previously required surgical intervention, particularly in MBO patients who are unsuitable for surgery.^[5]^ EUS-guided enterocolostomy combined with LAMS represents a novel technique that can be successfully used to treat MBO.^[6,7]^

In the present case, the patient had a multisegmental complex malignant intestinal obstruction with postoperative recurrence of rectal cancer, peritoneal metastasis, terminal ileum, and ascending colon obstruction and was unable to undergo surgery. Initial management included placement of a small bowel decompression catheter to relieve obstruction symptoms. Contrast medium was then infused through the small bowel decompression catheter to fill the ileum and facilitate identification of the target small bowel. Subsequently, a combination of EUS and fluoroscopic guidance was employed to identify the optimal puncture site. An electrocauterized LAMS (Hot AXIOS; Boston Scientific, Ballybrit Business Park, Galway, Irelang) was deployed from a puncture in the sigmoid colon to the ileum, achieving enterocolostomy [Figures 1–5; Video 1]. The patient’s obstructive symptoms were immediately relieved after surgery. The diet gradually transitioned to a semiliquid diet, and the patient was discharged after physical recovery. Three months after discharge, the patient reported 2–3 stools/d and received regular chemotherapy.

**Video 1.** 

The primary challenge of EUS-guided enterocolostomy is to accurately determine the site of intestinal obstruction and complete LAMS stent deployment by successfully crossing all intestinal obstruction segments.

**Figure 1.**
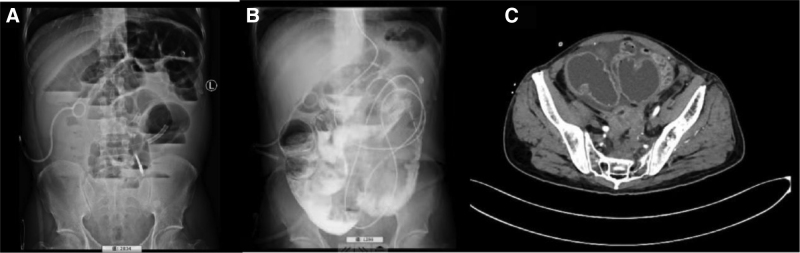
Multisegmental ileus and endoscopic enterocolostomy are possible. A, Small bowel decompression catheter placement to relieve obstruction symptoms. B, Gastrointestinal iodine contrast showing ileal and ascending colon multisegmental obstruction. C, CT showing pelvic ileal dilatation; endoscopic enterostomy is possible. CT, computed tomography.

**Figure 2.**
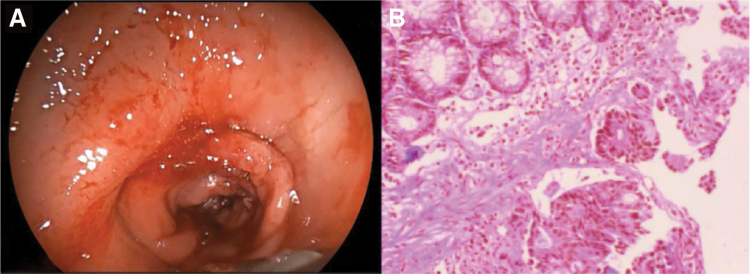
Recurrence and stricture after intestinal cancer surgery. A, Narrow intestinal tract 18 cm from the anus under endoscopy, preventing further insertion of the endoscope. B, Pathological indication of adenocarcinoma.

**Figure 3.**
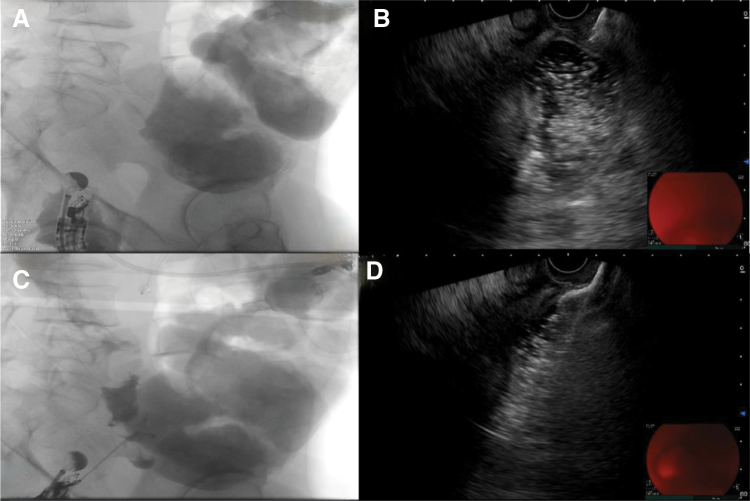
EUS-guided enterocolostomy. A, Contrast medium injected through the small bowel decompression catheter under fluoroscopy to fill the target small intestine; the endoscope was oriented to anchor the probe to the target small intestine 15 cm from the anus. B, Puncture from the sigmoid colon to the ileum using the integrated electrocauterized LAMS. C, Fluoroscopy to ensure correct puncture location. D, LAMS stent release. LAMS, lumen-apposing metal stent.

**Figure 4.**
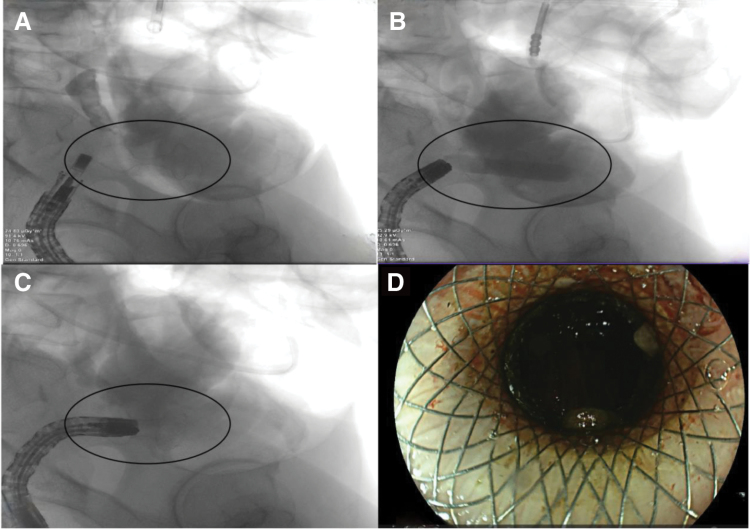
Step-by-step stent expansion with balloon (circled). A, X-ray fluoroscopy shows that the deployed stent is in good position, (B) use expansion balloon to expand the stent step by step, (C) X-ray fluoroscopy shows that the stent is well expanded, (D) endoscopy shows that the stent is well expanded.

**Figure 5.**
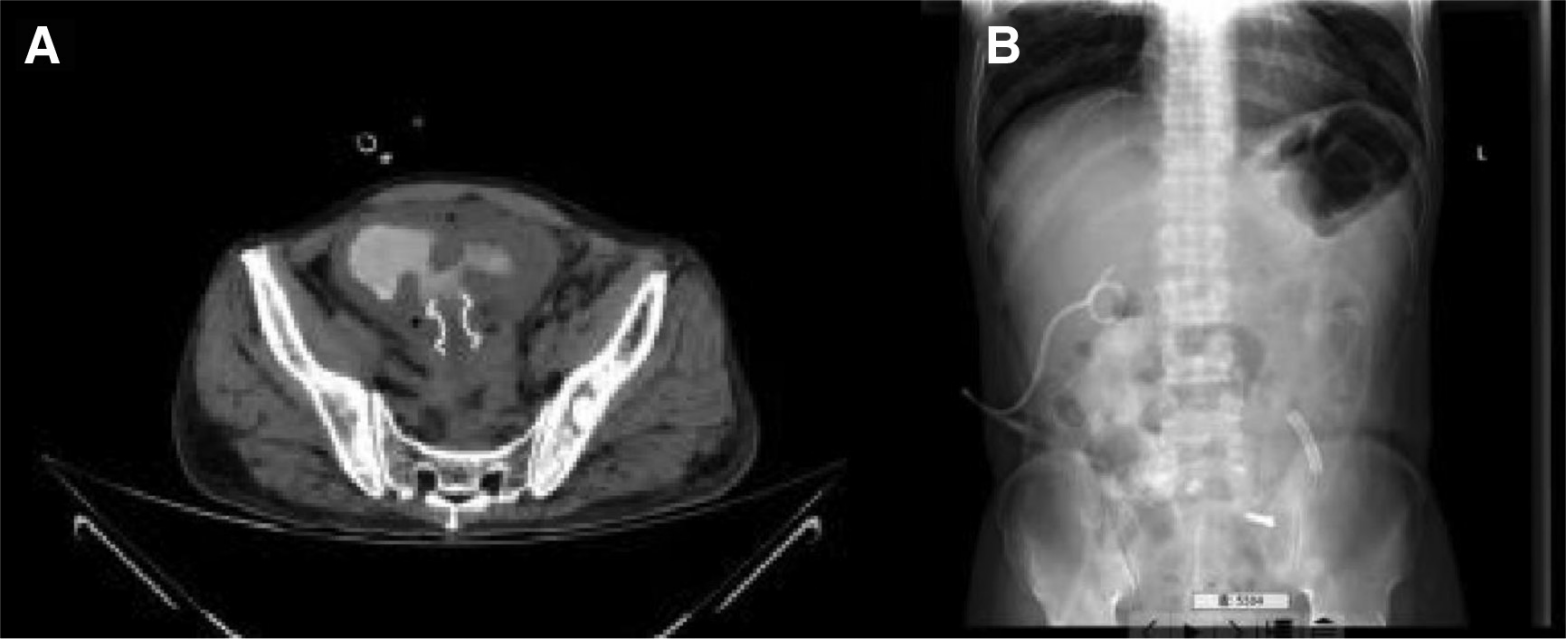
LAMS stent position is good, and postoperative symptoms are relieved. A, CT confirms the position of the LAMS stent. B, Abdominal plain film clearly shows obstruction relief. CT, computed tomography; LAMS, lumen-apposing metal stent.

## Supplementary Videos

Video 1: After thorough preparation and design of operation plan, we performed treatment after obtaining consent of family members. First, contrast medium was injected into small intestine through small intestine decompression catheter. Ultrasound endoscope combined with X-ray fluoroscopy repeatedly adjusted the position of ultrasound endoscope probe at sigmoid colon, and anchored the puncture point to the target intestinal tract at the upper end of obstruction. After determining the target small intestine, avoiding blood vessels, adjusting the shortest puncture path, puncture the intestinal wall with 19GFNA needle; The LAMS stent was released after contrast medium was injected into the lumen through the puncture needle to ensure correct puncture position. To fully expand the stent and ensure drainage patency, we used balloon to expand the stent. After expansion, suction was performed, and contrast medium was seen flowing out through the stent, further confirming the correct stent position. Videos are only available at the official website of the journal (http://www.eusjournal.com).

## Ethical Statements

The patient has signed the informed consent form. A single retrospective case report does not require ethical review.

## Conflicts of Interest

The authors declare that they have no conflict of interest with regard to the content of this report.

## Author Contributions

Sa. Fang and Y. Shi performed the endoscopy, Sa. Fang prepared the endoscopic figures and wrote the manuscript. M. Zhang collected medical history, S. Fang prepared the CT and X figures. J. Hao and X. Guo revised the manuscript carefully. All authors contributed to the care of the patient.

## Data Availability Statement

The datasets generated during and/or analyzed during the current study are available from the corresponding author on reasonable request.
